# Construction of Remote Sensing Model of Fresh Corn Biomass Based on Neural Network

**DOI:** 10.1155/2022/2844563

**Published:** 2022-05-31

**Authors:** Jianjian Chen, Hui Zhang, Yunlong Bian, Xiangnan Li, Guihua Lv

**Affiliations:** ^1^Institute of Maize and Featured Upland Crops, Zhejiang Academy of Agricultural Sciences, Dongyang, Zhejiang 322100, China; ^2^Zhejiang Agricultural Technology Extension Center, Hangzhou, Zhejiang 310000, China; ^3^Jiangsu Key Laboratory of Crop Genetics and Physiology, Co-Innovation Center for Modern Production Technology of Grain Crops, Key Laboratory of Plant Functional Genomics of the Ministry of Education, Yangzhou University, Yangzhou, Jiangsu 225009, China

## Abstract

Corn has a high yield and is widely used. Therefore, developing corn production and accurately estimating corn biomass yield are of great significance to improving people's lives, developing rural economy and climate issues. In this paper, a 3-layer BP neural network model is constructed by using the LM algorithm as the training algorithm of the corn biomass BP network model. From the three aspects of elevation, slope, and aspect, combined with the BP neural network model of corn biomass, the spatial distribution of corn biomass in the study area is analyzed. The results showed that the average biomass per unit area of maize increased with the increase in altitude below 1000 m. There are relatively more human activities in low altitude areas, which are more active in forestry production. The best planting altitude of corn is 0 ∼ 1000 m. When the altitude is higher than 1000 m, the corn biomass gradually decreases. In terms of slope, if the slope is lower than 15°, the biomass of maize increases with the increase in slope. If the slope is lower than 15°, the biomass of maize decreases gradually with the increase in slope. The biomass of maize on sunny slope was higher than that on shady slope.

## 1. Introduction

Corn is an important food crop. Corn has strong adaptability, wide distribution, multiple uses, and great potential for increasing production. It ranks third in the world in terms of sown area and total output after rice and wheat and is developing rapidly. As an important food crop, corn is also rich in nutrients [1–3]. Since corn resources are extremely abundant, cheap, and easy to obtain, they also have many biological activities, such as antioxidation, antitumor, hypoglycemic, improving immunity, and bacteriostasis, which have broad development and application prospects. Therefore, developing corn production and accurately estimating corn biomass yield are of great significance to improving people's lives, developing rural economy, and solving climate issues [4–6].

Remote sensing information model method has become one of the most important methods in biomass research. This method estimates forest biomass through the correlation between chlorophyll content information reflected in remote sensing images and forest biomass. Therefore, the correlation mathematical model between remote sensing spectral information and measured biomass was established, and the forest biomass was estimated by inversion [7]. The remote sensing information model method is not only suitable for biomass estimation in small areas but also shows good results in biomass estimation in large areas. According to the different mechanisms of using remote sensing to estimate forest biomass, it can be divided into multiple regression analysis fitting relationship method, artificial neural network method, and so on [8–10].

The correlation analysis between the vegetation index and crop physiological and biochemical parameters established by the reflectance of different bands of the crop canopy can build a remote sensing biomass estimation model under different conditions and realize the remote sensing estimation of crop biomass yield [11, 12]. The current sensors for constructing such characteristic bands or vegetation indices are multispectral [13], visible RGB camera [14], and hyperspectral [15]. The relationship models established by using canopy spectral information mainly include linear (univariate and multivariate) regression models, nonlinear (exponential, logarithmic) regression models, powerful machine learning regression algorithms, and deep learning algorithms [16, 17]. Because there is a strong relationship between plant biomass and its own physical structure information (height, leaf area, bulk density, etc.) Therefore, biomass estimation models can be established through canopy structure parameters, such as tree allometric models [18] and regression models [19].

The measurement of crop canopy structure parameters relies on detecting the true location of the crop canopy and the terrain below the canopy, which means that a digital surface model that accurately represents the terrain is required to derive the vegetation canopy structure [20]. At present, the main sensors used to obtain the three-dimensional structure information of crop canopy based on remote sensing platform are visible light RGB camera and lidar LiDARo RGB camera. Compared with other sensors, the cost is lower and the time resolution is higher. The consumer-grade UAV RGB system can obtain high-precision images, construct crop surface models and digital elevation models, and generate orthophotos, point cloud information, and so on to determine crop structure information through image processing [21] to establish a data basis for accurate estimation of crop biomass [7, 22, 23].

Bendig et al. [24] used an airborne RGB camera to estimate the average canopy height and then estimated the fresh biomass (*R*^2^ = 0.81) and dry biomass (*R*^2^ = 0.82) of barley through the established exponential model. Ballesteros et al. [25] used RGB camera-based estimates of vegetation cover, plant height, and canopy volume to evaluate dry leaf biomass and dry bulb biomass of onion with *R*^2^ of 0.76 and 0.95, respectively. Liu et al. [26] estimated the aboveground biomass based on the visible light spectral information and texture features of the potato canopy at different heights. The results show that adding texture features can greatly improve the damage estimation accuracy. LiDAR has high spatial resolution, is not affected by ambient light, and is highly repeatable, so it can provide more accurate three-dimensional structure information of vegetation canopy at the field scale than RGB cameras [27–31].

The advantage of BP neural network lies in its strong fitting ability, especially in biomass remote sensing inversion. The BP model can use the brightness values of multiple spectral bands of multispectral data (such as TM, ETM+, and so on). The underlying regularity between forest biomass and its spectral reflectance properties can be accurately estimated [32]. The research by Guo et al. [33] shows that the BP model shows superior characteristics in remote sensing inversion of forest biomass. Among them, Guo et al. [33] established a biomass remote sensing model based on neural network technology using Xiaoxing'anling TM images combined with ground survey data, with high accuracy. Their research also used conventional methods to establish regression models and compared the two methods. The analysis points out that the mechanism between biomass and remote sensing factors in the regression model of the conventional method is easy to elucidate, while the estimation accuracy of the neural network method model is higher [12–15, 17].

Based on the BP neural network platform, a nonlinear model system of forest biomass remote sensing is established. The relative errors of this model system in estimating the biomass of coniferous forest, broad-leaved forest, and mixed coniferous and broad-leaved forest are −1.147%, 2”38%, and 3.156%, respectively. The estimation accuracy is high. And we use the model system to generate the quantitative distribution map of biomass in the study area. The overall accuracy reaches 88%. The use of the geographic information system (GIS) and remote sensing images combined with ground information to estimate biomass has been able to estimate the amount of mass in a large area. Artificial neural network has good nonlinear approximation ability and plays an important role in the establishment of multidimensional nonlinear models. It can establish nonlinear remote sensing models with high fitting accuracy, making the types of remote sensing models more abundant. Wang et al. [34] established a nonlinear remote sensing model system based on a neural network platform and used this model to estimate the biomass of coniferous forest, broad-leaved forest, and mixed coniferous and broad-leaved forest in the study area [20, 32, 35, 36]. The overall estimation accuracy reached 88%. In this paper, based on neural network, the biomass remote sensing model of fresh corn is constructed and analyzed.

## 2. Methods and Theory

### 2.1. Artificial Neural Network

Artificial neural network is a new theoretical method that has emerged in recent years. It is a type of model established by simulating the human brain nervous system from the microscopic structure and function. It is a way to simulate human intelligence. Compared with other methods, it has the following characteristics: (1) does not require preassumption, only needs to learn sample training; (2) can be well suited to the advantage of noisy data. This kind of network depends on the complexity of the system and achieves the purpose of processing information by adjusting the interconnected relationship between a large number of internal nodes. Nonlinear adaptive information processing system consists of a large number of interconnected processing units. It is proposed on the basis of modern neuroscientific research results and attempts to process information by simulating the neural network of the brain to process and memorize information. The artificial neural network has the ability of self-learning and self-adaptation. It can analyze and grasp the potential rules between the two through a batch of corresponding input-output data provided in advance and finally use the new input data to calculate the output according to these rules.

Artificial neural network is a nonprogrammed, adaptive, brain-style information processing. Its essence is to obtain a parallel and distributed information processing function through the transformation and dynamic behavior of the network and to imitate human beings to different degrees and levels. It is an interdisciplinary subject involving neuroscience, thinking science, artificial intelligence, computer science, and other fields and has a wide range of applications in various industries.

### 2.2. Model Construction

#### 2.2.1. Selection of Independent Variables

The brightness value of remote sensing images fully reflects the spectral characteristics of ground objects. The visible light band (Band 1–3), near-infrared band (Band 4), and short-wave infrared band (Band 5, 7) of ETM +  re useful in vegetation identification and forest growth prediction. The derived bands of ETM + images, namely, vegetation index, are also commonly used data in plant classification and identification. From the perspective of ecology and geology, elevation, slope, and aspect also affect the growth of plants. Therefore, the selected modeling independent variables include 3 categories: ETM + original bands (Band 1–5, 7), derived bands of ETM + data (vegetation index DVI, normalized vegetation index NDVI, and ratio index RVI), and geoscience information (DEM, SLOPE, and ASPECT).

The vegetation index is based on the different reflection characteristics of different vegetation to red light and near-infrared light, and the image operation of these two bands is carried out, so as to enhance the vegetation information and weaken the useless information, which has achieved the purpose of vegetation identification and plant biomass prediction. The calculation methods of each vegetation index are as follows:

Difference vegetation index is as follows:

(1) $$\begin{array}{c} \text{DVI}={\text{DN}}_{\text{NIR}}-{\text{DN}}_{R}. \end{array}$$

DN_NIR_ and DN_R_ represent the brightness values of ETM + near-infrared band and red light band, respectively.

Ratio vegetation index is as follows:

(2) $$\begin{array}{c} \text{RVI}=\frac{{\text{DN}}_{\text{NIR}}}{{\text{DN}}_{R}}. \end{array}$$

Normalized vegetation index is as follows:

(3) $$\begin{array}{c} \text{NDVI}=\frac{\left({\text{DN}}_{\text{NIR}}-{\text{DV}}_{R}\right)}{\left({\text{DN}}_{\text{NIR}}+{\text{DN}}_{R}\right)}. \end{array}$$

The slope and aspect are obtained by performing terrain analysis on DEM in ArcGIS 9.3 software. In addition, the location data of various places are obtained in the field through GPS. Due to the influence of terrain and other factors, the location data will have certain errors. In order to eliminate the errors caused by GPS positioning data, a neighborhood analysis of 3 × 3 pixels is performed on each independent variable; that is, the value of each pixel is replaced by the average value of 9 surrounding pixels (including itself). There is a great correlation between vegetation index, original zone, altitude, slope direction, and original zone. Therefore, principal component analysis was performed on 12 variables, and the autocorrelation between the variables was eliminated by dimensionality reduction and finally compressed into 8 principal components, which were used as the input variables of the model. Similarly, from the correlation between biomass and their respective variables, it can be seen that the linear correlation between biomass and each factor is not high, indicating that the relationship between biomass and remote sensing factors and geoscience factors should not be explained by a linear relationship, which should be explained by the BP neural network model with strong nonlinear fitting ability.

#### 2.2.2. Construction of the BP Model

**Figure 1:**
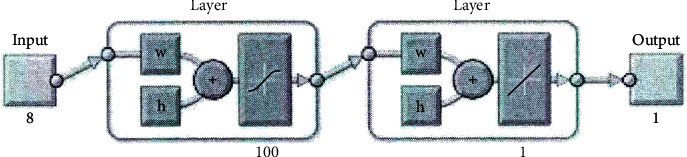
Diagram of BP network model structure.

We construct a 3-layer BP neural network model (i.e., an input layer, a hidden layer, and an output layer), in which the number of nodes in the input layer is 8, the number of nodes in the hidden layer is 100, and the number of nodes in the output layer is 1. In this paper, the LM algorithm is used as the training algorithm of the forest biomass BP network model. The remote sensing factor data, terrain factor data, and biomass data of 40 plots are used as the training samples of the model, and 8 principal components obtained by principal component analysis of 12 original variables are used as model input variables. However, due to the dimension of each factor, the size and range of the data are different. The original data are normalized and then used for modeling to prevent the small data information from being overwhelmed by the large data information. The final simulation result is restored by denormalization processing. The biomass of the plot was normalized as the output variable of the model. According to the characteristics of the data, the hyperbolic tangent function tansig is selected as the transfer function from the input layer to the hidden layer, and its value range is (−1, 1), so as to ensure that the input value of the hidden layer is between (−1, 1). The linear function purelin is used as the transfer function from the hidden layer to the output layer. The structure of the BP model is shown intuitively in Figure 1. In the figure, *W* is the weight matrix and *b* is the threshold matrix.

## 3. Results and Discussion

### 3.1. Evaluation of the BP Model

**Figure 2:**
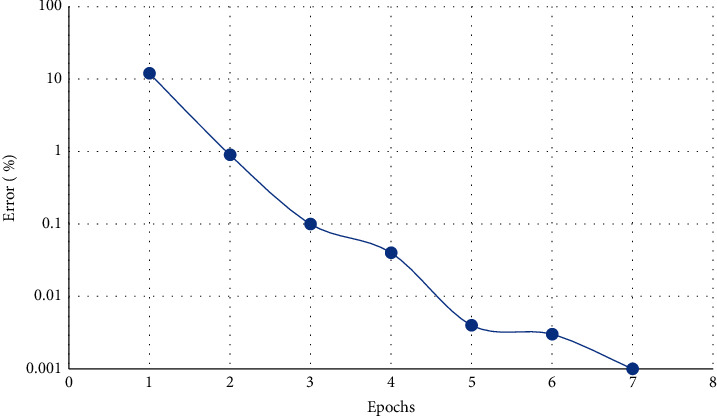
Variation of training error of the fresh corn biomass network model.

**Figure 3:**
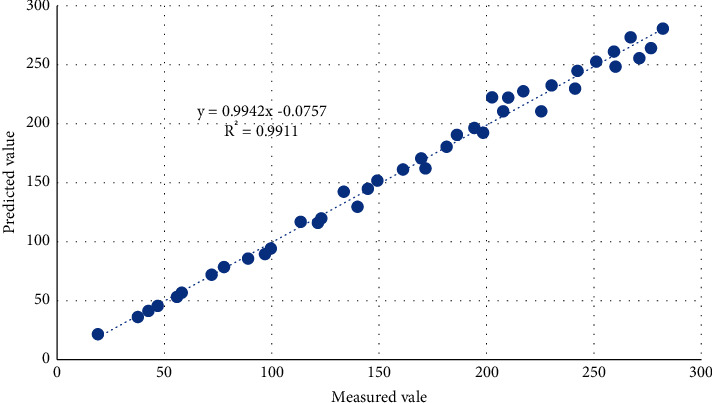
Fitting effect of the fresh corn biomass network model.

Figure 2 shows the change of the mean square error during the training process of the BP model. When epoch = 7, it reaches the preset minimum error of 0.001 (the error here is the normalized error term).  Figure 3 is the fitting effect diagram of the BP neural network model. The predicted value is highly correlated with the measured value, indicating that the fitting effect of the model is ideal.

### 3.2. Spatial Distribution of Biomass

Previous practice and research show that the distribution of fresh corn is significantly related to climate and topographic changes. As a typical species, the distribution of maize shows obvious regularity with the change of terrain, and the spatial distribution of fresh maize biomass is also different. Combined with the BP neural network model of fresh corn biomass, the spatial distribution of corn biomass in the study area was analyzed from three aspects: altitude, slope, and slope direction.

#### 3.2.1. Altitude

**Figure 4:**
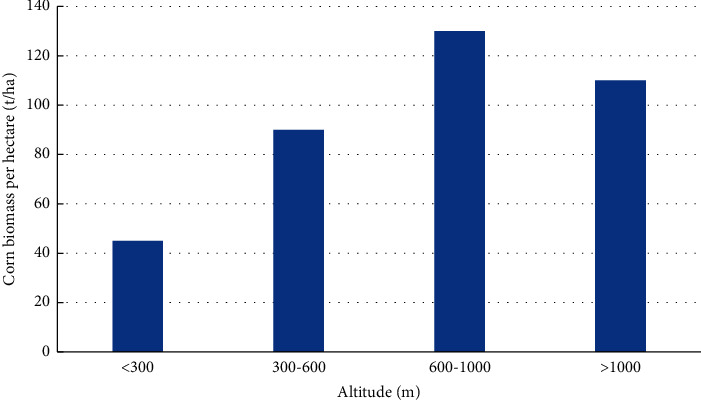
Altitude distribution of corn biomass in the study area.

It can be seen from Figure 4 that the average fresh corn biomass per unit area shows a gradual upward trend with the increase in altitude. Compared with the area below 300 m above sea level, the fresh corn biomass per unit area increased significantly in the area of 300–600 m above sea level, which was related to human production activities. Low-elevation areas tend to be areas with relatively frequent human activities, areas where forestry production is more active, so fresh corn biomass levels are high. Conversely, in areas with relatively high elevations, i.e., 300–1000 meters, the corn quality in this area is higher and the biomass level is lower due to the reduced accessibility of corn fields and relatively less anthropogenic disturbance. In areas above 1,000 meters above sea level, corn biomass levels decreased, possibly due to a decrease in quality due to decreased moisture and climatic conditions due to increased altitude.

#### 3.2.2. Slope

The energy exchange and material circulation of fresh corn ecosystems are obviously affected by the slope. Some scholars have pointed out that the solar radiation energy, soil bedrock, soil organic matter content, corn water holding capacity, and so on in the ecosystem all show variability within different slope ranges. In addition, increasing the slope can limit human movement. All these reasons lead to different distribution laws of corn biomass on different slopes. The slope is divided into five grades: 0–10° is a flat slope, 10–15° is a gentle slope, 15–30° is a normal slope, 30–45° is a steep slope, and above 45° is a steep slope.

**Figure 5:**
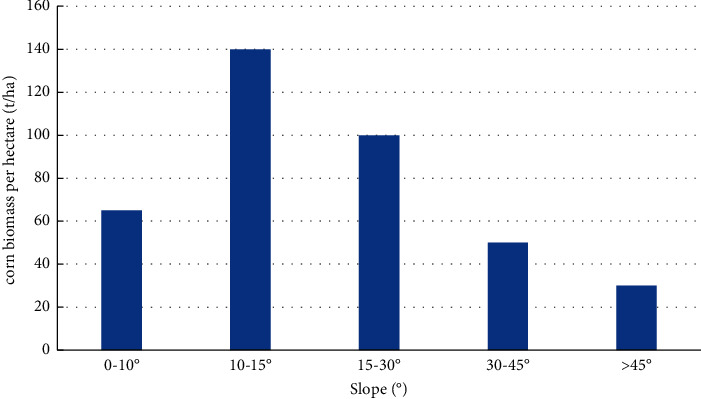
Variation of corn biomass level with slope.

It can be seen from Figure 5 that the average corn biomass per hectare is only 65 t/ha in the flat slope area with a slope of less than 15°. Gentle slopes and slopes are areas with high corn biomass levels, with an average of 140 t/ha for gentle slopes and 100 t/ha and for slope areas and steep slopes, the biomass level decreased sharply. The change trend of biomass level with slope can be attributed to several reasons. First, most of the flat slope areas are at lower altitudes, where human activities are the most frequent and corn is greatly affected by human disturbance and human production activities, so the quality of corn is not high. The biomass level is low. Secondly, gentle slopes and normal slope areas are usually the areas that the government and forest farmers pay more attention to, and they are the objects that people focus on tending and protecting, and as the slope increases, the corn is less damaged by humans, so corn grows. As the slope continued to increase, the climatic conditions decrease, the surface interception decreases, and the ability of corn to maintain soil and water decreases, resulting in the decline of forest quality and the gradual decline of the corn biomass level.

#### 3.2.3. Slope Aspect

**Figure 6:**
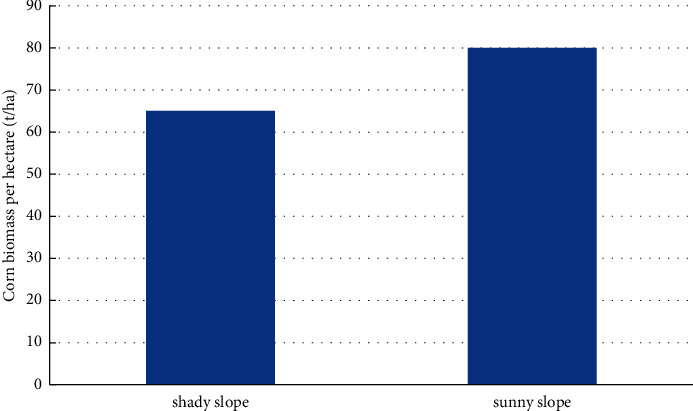
Variation of corn biomass level with slope direction.

Under the same slope and altitude, slope aspect is a factor that has an obvious impact on corn distribution and corn biomass also varies significantly with slope aspect. It is evident from Figure 6 that corn grown on sunny slopes had higher biomass levels than corn grown on shady slopes.

## 4. Conclusions

1. The LM algorithm is used as the training algorithm of the corn biomass BP network model, and a 3-layer BP neural network model is constructed. Among them, the hyperbolic tangent function is selected as the transfer function from the input layer to the hidden layer, the value range is (−1, 1), and the linear function is selected as the transfer function from the hidden layer to the output layer, without any transformation of the output value. By fitting the effect diagram of the BP neural network model, the results show that the predicted value is highly correlated with the measured value, indicating that the fitting effect of the model is ideal.
2. We input the image data after principal component transformation and normalization into the BP neural network model, from the three aspects of altitude, slope, and slope aspect, combined with the BP neural network model of corn biomass, to analyze the distribution of corn biomass in the study area. The results showed that when the altitude was lower than 1000 m, the average biomass per unit area of maize increased with the increase in altitude, and when the altitude was higher than 1000 m, the average biomass per unit area of maize decreased rapidly with the increase in altitude. In terms of slope, in the flat slope area with slope less than 15°, the average biomass of corn per hectare increases rapidly with the increase in slope, and the biomass level of slope is higher than that of steep slope, and the steep slope is unstable. With the increase in slope, the biomass level shows a sharp downward trend. The biomass of maize on sunny slope was higher than that on shady slope.
3. The training algorithm of the BP network model is suitable for the construction of the remote sensing model of fresh corn biomass.

## Data Availability

The figures and tables used to support the findings of this study are included in the article.
